# Improving selection procedures in health professions education from the applicant perspective: an interview study

**DOI:** 10.1186/s12909-024-05761-z

**Published:** 2024-08-07

**Authors:** S. Fikrat-Wevers, K. M. Stegers-Jager, L. M. A. Mulder, J. Cheung, W. W. Van Den Broek, A. M. Woltman

**Affiliations:** 1 Institute of Medical Education Research Rotterdam, Erasmus MC, University Medical Center Rotterdam, Room Na2310, PO Box 2040, 3000 CA Rotterdam, the Netherlands; 2 Amsterdam UMC location Vrije Universiteit Amsterdam, Research in Education, Amsterdam, The Netherlands

**Keywords:** Selection, Admission, Student diversity, Applicant perceptions

## Abstract

**Introduction:**

Applicant perceptions of selection impact motivation and performance during selection, and student diversity. However, in-depth insight into which values underly these perceptions is lacking, creating challenges for aligning selection procedures with applicant perceptions. This qualitative interview study aimed to identify values applicants believe should underlie selection, and how, according to applicants, these values should be used to make specific improvements to selection procedures in undergraduate health professions education (HPE).

**Methods:**

Thirty-one applicants to five undergraduate HPE programs in the Netherlands participated in semi-structured interviews using Appreciative Inquiry, an approach that focuses on what goes well to create vision for improvement, to guide the interviews. Transcriptions were analyzed using thematic analysis, adopting a constructivist approach.

**Results:**

Applicants’ values related to the aims of selection, the content of selection, and the treatment of applicants. Applicants believed that selection procedures should aim to identify students who best fit the training and profession, and generate diverse student populations to fulfill societal needs. According to applicants, the content of selection should be relevant for the curriculum and profession, assess a comprehensive set of attributes, be of high quality, allow applicants to show who they are, and be adapted to applicants’ current developmental state. Regarding treatment, applicants believed that selection should be a two-way process that fosters reflection on study choice, be transparent about what applicants can expect, safeguard applicants’ well-being, treat all applicants equally, and employ an equitable approach by taking personal circumstances into account. Applicants mentioned specific improvements regarding each value.

**Discussion:**

Applicants’ values offer novel insights into what they consider important preconditions for the design of selection procedures. Their suggested improvements can support selection committees in better meeting applicants’ needs.

**Supplementary Information:**

The online version contains supplementary material available at 10.1186/s12909-024-05761-z.

## Background

Due to the high stakes involved, selection into undergraduate health professions education (HPE) is a topic of public and academic debate [1]. Nevertheless, consensus on the optimal design of selection procedures is currently lacking. In a quest for the most appropriate methods to select successful and diverse student cohorts to meet societal needs, significant efforts have been invested in researching the predictive validity of selection methods and their impact on student diversity [2, 3]. While incorporating input from stakeholders has been shown to be another important step for shaping selection procedures [1], one crucial perspective has remained relatively unexplored: that of the applicants themselves. Although previous studies have investigated applicant perceptions of existing selection tools, little attention has been paid to understanding applicants’ viewpoints regarding what constitutes an ideal selection procedure. By acknowledging applicants as active and eminent stakeholders in designing the selection procedure, this study seeks to address this gap in the literature. In the present qualitative study, we investigated what values applicants believe should underlie selection procedures and how applicants believe these values can be translated into specific improvements of selection procedures in undergraduate HPE. Values, in this context, can be defined as the standards that determine whether something is being perceived as desirable or not [4, 5]. Hereby, our study provides an in-depth understanding of applicant perceptions, facilitating the integration of their needs into the design of selection procedures.

The design and development of selection procedures should be informed by feedback from stakeholders [1]. In this regard, taking applicant perceptions into account is particularly important, as their perceptions can have practical impact on applicants at multiple stages. First, perceptions of the selection procedure could impact the decision to apply for an HPE program; when applicants have negative perceptions of or are deterred by the selection procedure, they can shy away from applying [6, 7]. Additionally, perceptions can have consequences for applicant motivation and performance, and negative perceptions can even cause withdrawal from the selection procedure once applicants are undergoing the selection procedure [8–11]. Notably, this can impact the ability of selection to admit a diverse student cohort, since applicants from lower socioeconomic and ethnic minority backgrounds tend be more susceptible to negative perceptions of selection, which is often due to the experiences of unequal access to resources, such as commercial coaching activities [1, 6, 12]. In addition, applicants from underrepresented backgrounds may underestimate their selection chances [12, 13].

Even though the importance of understanding applicant perceptions regarding selection for jobs and schools is evident, research about this topic in HPE is currently focused on perceptions regarding existing individual methods (e.g., grades, interviews). Most of these studies focus on one specific method [14], whilst some incorporate a comparative design. Such comparisons concluded that applicants prefer selection methods such as curriculum-sampling tests, skills tests and interviews over cognitive tests, grades and different types of lottery [1, 15, 16]. This suggests that applicants prefer selection methods through which they (1) can demonstrate skills beyond their cognitive abilities and (2) feel more “in control” [15]. However, it is unknown what specific needs and values underlie their preferences for particular methods, which requires qualitative research [1]. Improved understanding of such underlying mechanisms is needed for two reasons. First, applicant perceptions regarding selection methods tend to contradict evidence from other indicators of validity [1, 11]. Grades, for instance, are often perceived as unfavorable [11, 15], while their predictive validity is evident [17]. Second, applicants themselves often express contradictory perceptions of what is appropriate for selection [15]. For instance, in previous research, applicants expressed the wish to combine multiple selection methods for completeness, whilst they also warned that a combination of methods could induce too much stress [15]. That study revealed that applicants can provide valuable insights into issues that could remain overlooked by educational institutions. Nevertheless, another notable contradictictory perception identified by that study is that even though applicants believe currently used selection methods can hinder equitable admission to HPE due to a perceived unequal access to resources for preparation [6, 12, 15, 18], applicants still prefer performance-based selection methods over an unweighted lottery that could counter the negative effects of unequal access to resources [15]. Due to the aforementioned contradictions, the practical usability of previous insights into applicant perceptions remains limited. Moreover, the prior focus on the evaluation of individual methods provoked a negative, problem-centered approach to the understanding of applicant perceptions [15].

Thus, a gap in the applicant perceptions literature exists considering research that views applicants as active stakeholders with valuable input rather than solely focusing on perceptions of existing selection methods. In addition, there is a need for a more in-depth and positive grasp of applicants’ views and attitudes regarding selection, in order to better take these into account in the design of selection procedures [1]. Given the lack of prior research in this area, it is intriguing to initiate an initial exploration into actively involving applicant in the selection procedure's design by examining the values that applicants believe should underlie selection. As values underly perceptions [4, 5], they must be congruent between selection and applicant, in order for a selection procedure to be perceived as acceptable. To our knowledge, however, no research has been conducted to identify those underlying values. Insights into these values can provide new directions for improving the selection procedures of undergraduate HPE programs. Therefore, the aim of the present study was to identify preconditions for improving selection procedure, derived from applicants’s perceptions of what values should be the basis for selection procedures in undergraduate HPE. We aimed to accomplish this objective by (1) identifying the values that selection committees should adopt from the perspective of applicants when establishing the foundations of the selection of prospective students in HPE, and (2) understanding how applicants believe that their underlying values should be used to make specific improvements to the selection procedure.

## Methods

### Design and context

The design of the present study was drawn upon constructivist philosophy. We designed a cross-sectional study, in which one-on-one interviews were conducted with a diverse group of applicants. As the idea within constructivism is that meaning can be constructed in the researcher-participant interaction, interviews can provide in-depth data [19]. The sample of the present study consisted of applicants who participated in the selection procedures of five undergraduate HPE programs in the Netherlands: three medical programs, one technical-medical program and one pharmacy program. The included programs were located in different parts of the Netherlands, both in urban and rural areas, and were all concerned with improving their selection procedures.

One distinctive feature of the Dutch educational system is that admission requirements for different types of undergraduate HPE programs are identical. To be eligible, applicants need to meet the same stringent requirements regarding subjects taken (e.g., physics, chemistry, and biology) and educational level (i.e., graduation level of pre-university education). Although applicants can apply from different educational routes, they all need to provide proof that they meet aforementioned requirements. Consequently, the applicant pools are relatively homogeneous in terms of academic background; students who apply to a university-level undergraduate HPE program are already strongly preselected based on academic skills due to highly selective secondary education [20]. When applicants apply to their program of choice, they apply to one specific institution. Each institution has a predetermined fixed number of spots. At the time of this research, Dutch institutions were required to incorporate a minimum of two qualitative selection criteria in their selection procedure, but there were no additional requirements regarding, for instance, the content and quality of the selection methods. Consequently, great variety exists in the selection procedures that programs employ, both between and within different types of HPE programs at different institutions.

The five included programs had different self-designed selection procedures, which are summarized in Additional file 1. More information about the selection procedures (including number of applicants and acceptance rates) can be found elsewhere [21]. Noteworthy is that equitable admission policies (e.g., holistic or contextualized admissions) were not permissible in the Netherlands. The wide range of HPE programs and selection procedures provides us with a diverse set of experiences and perceptions of applicants, contributing to a more comprehensive understanding of their values.

### Procedure

Participants were recruited during the selection procedures for the academic year 2021-2022. Recruitment letters were sent by email to all applicants in the selection procedures of the five participating HPE programs. We aimed to include at least five participants from each program, to ensure representation of a diversity of experiences and perspectives in the data. Aside from participating in the selection procedure of at least one program, no other inclusion criteria were applied. However, we did aim to generate a diverse group of participants to represent different perspectives on the topic. Since a large number of applicants was interested in participating in the study, we used purposive sampling to compose a diverse group of participants in terms of gender and ethnic background. This was done by looking at the names of interested applicants, and specifically including applicants who appeared to be members of groups underrepresented in HPE in our invitations. Recruitment and selection of participants was done by SFW.

The interviews were conducted between February 2021 and April 2021, after the applicants had participated in the selection procedure but before the selection outcomes were communicated. This way, applicants had a sufficient understanding of what selection into an HPE can entail, but their perceptions would not be affected by the selection outcomes. Based on previous research [22], we expected that approximately 30 interviews would be necessary to reach data sufficiency, meaning that additional interviews would not yield new insights into the research topic [23]. This was indeed the case. Due to COVID-19, interviews took place via an online one-on-one video call. The interviews lasted for 30-60 minutes and were conducted by SFW. The interviewer was not involved in any of the selection procedures and had no relationship with the participants.

### Interviews

Interviews were semi-structured, with questions derived from the principles of Appreciative Inquiry [24]. Appreciative Inquiry is an approach that focuses on identifying what goes well and envisioning what would work well in the future, with the goal of using those strengths to drive positive change and transformation [24]. It offers a possibility to thoroughly examine applicants’ values regarding selection, whilst creating a vision for change [24]. A certified Appreciative Inquiry expert was involved in the design of the interview guide. At the beginning of each interview, SFW introduced herself, tried to make the applicants at ease and explained the purpose of the interview. The interview consisted of four main questions, based on the 4-D cycle of Appreciative Inquiry methodology as described by Sandars and Murdoch-Eaton [24]. The 4-D cycle encompasses Discovery (identifying what goes well), Dream (envisioning an ideal future), Design (planning an ideal future) and Destiny (executing the proposed design). Destiny was left out from the interview, because applicants do not have any influence on the execution the design of the selection procedure. The full interview guide can be found in Additional file 2.

At the end of the interview, participants were asked to fill out a form with the following demographic characteristics: age, gender, prior education, parental education level, and ethnic background. Gender diversity was acknowledged in the present study, and applicants had the option to choose between three categories: ‘man’, ‘woman’ and ‘other, namely [free text box]’. With respect to prior education, we distinguished between standard Dutch pre-university education, university, higher vocational education and other (including a free text box). Parental education was used as a proxy for socioeconomic status, and applicants were categorised as first-generation university applicants when none of their parents had attended higher education (university or higher vocational education). Finally, the item on ethnic background was a free text box in which applicants could provide their ethnic identity. This form was administered to ensure that a diverse group of applicants was included in our sample in order to prevent blind spots in our data.

### Data analysis

The interviews were analyzed using a social constructivist paradigm, with the central belief that reality is subjective and can be interpreted in different ways [19]. Since we aimed to take the individual experiences of participants as a starting point, we used inductive reasoning to interpret the meanings of participants’ responses [25]. We used thematic analysis to find patterns in the data, because this method is considered useful to analyze experiences, perceptions and thoughts [26]. The six-step framework for thematic analysis as described by Kiger and Varpio was used to guide the analyses [26]. In the case of our study, the themes we aimed to identify were values. Therefore, in the rest of the paper, we will use the term values instead of themes.

SFW read the interview transcripts multiple times to familiarize herself with the data. To generate initial codes, SFW, LM and JC all independently conducted open coding of 5 interviews, after which differences in coding were discussed until consensus. Based on this initial coding, SFW developed a coding manual. Subsequently, SFW coded all interviews in Atlas TI version 22, with JC coding a subsample of the interviews. Consistency checking of the coding was done by KSJ, LM and AW: KSJ and AW checked a subset of interviews, whilst LM checked a subset of codes. SFW constructed initial values based on the coding, and reviewed, refined and defined the final values. In this process, all authors (SFW, KSJ, LM, JC, WVDB & AW) critically reflected on the values at several stages, including their fit with the data. Once the values were finalized, SFW returned to the codes to match the changes proposed by applicants to the values. Usually, the proposed changes were lower order codes that could easily be linked to higher codes related to the values. In some cases, it was necessary to reread the transcripts to contextualize the suggestions for improvement. Finally, SFW reported the results with the assistance of KSJ and AW.

The research team shared a variety in professional and demographic backgrounds. SFW and KSJ have a background in educational sciences, LM in sociology, JC is a medical student who had undergone a medical school selection procedure himself, WVDB has a background in medicine and educational management, and AW in biomedical sciences and educational management. This led to a diversity of perspectives and contributed to reflexivity and critical dialogue throughout the analytical process, ensuring interpretation of data using different conceptual lenses. An example of reflexivity is that we first formulated a value about equality, but during our discussion realized that certain comments of students that were defined as equality in fact described equity. Consequently, we reconsidered this value and divided it into two values.

## Results

### Participants

In total, 31 applicants were interviewed, with at least five applicants of each program. All participants filled out the demographics survey. One third of the interviewed applicants identified as men (*N*=10; Table 1), two-thirds identified as women (*N*=20) and one applicant identified as non-binary. Twenty-one applicants were graduating from year six of pre-university education, while eight applicants had already pursued another type of higher education and two applicants had an alternative form of prior education. Around a quarter of the applicants (*N*=8) were first-generation university applicants. Finally, around half of the interviewed applicants identified with another ethnic background than Dutch (*N*=15), with a wide range of backgrounds. Distributions of demographic variables were similar to previous research within the same target group, although applicants with an ethnic minority background were overrepresented due to purposive sampling in the present study [15, 21].

**Table 1:** Participants’ background characteristics

Gender	Man	10
	Woman	20
	Non-Binary	1
Prior education	Pre-university education	21
	University	5
	Higher vocational education	3
	Other	2
Socioeconomic background	No first-generation university applicant	23
	First-generation university applicant	8
Ethnic background	Ethnic majority background	16
	Ethnic minority background	15
Program^a^	Medicine, Amsterdam UMC, location AMC	6
	Medicine, Erasmus MC	7
	Medicine, Amsterdam UMC, location VUmc	6
	Pharmacy, Utrecht University	7
	Technical Medicine, University of Twente	5

^a^One student participated in two selection procedures, i.e., Medicine at Erasmus MC and Pharmacy at Utrecht University

### Values underlying selection into health professions education

**Fig. 1:**
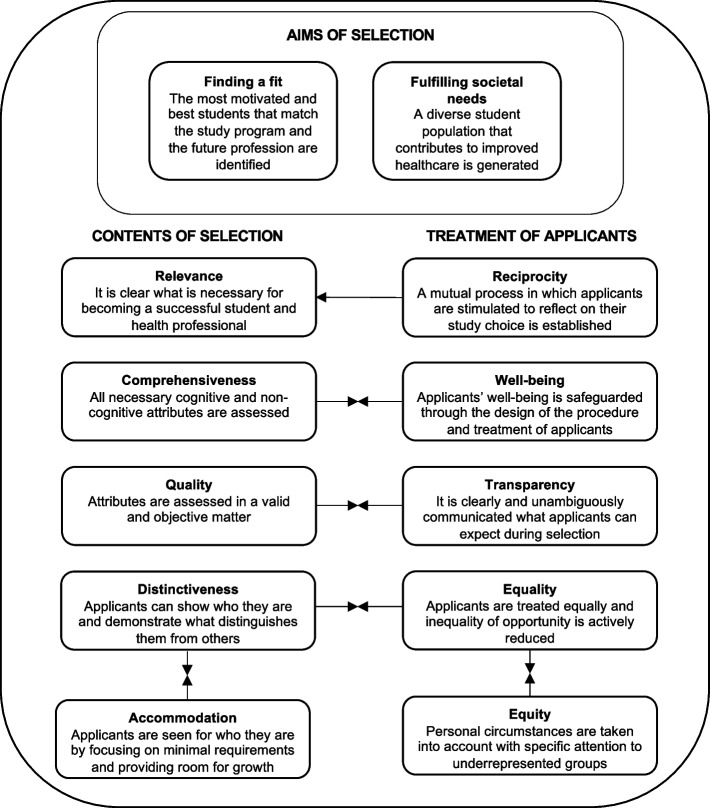
Thematic map summarizing the values that emerged from the data. Legend. Two perpendicular arrows indicate a friction between two values

The values that were discussed in the interviews could be grouped into three categories. The category 'Aims of selection’ comprises of what applicants think the selection should aim for, whereas ‘Contents of selection’ is about characteristics of the selection itself, and ‘Treatment of applicants’ deals with how applicants wish to experience the selection procedure. Ideally, the latter two categories contribute to the ‘Aims of selection’. In the next sections, an explanation of each value will be provided, as well as examples of specific suggestions for improvements, and, if applicable, an explanation of potential frictions between values (Figure 1). Most values related to broader societal values, whilst some values related more specificly to the context of health professions education.

### Aims of selection

#### Finding a fit

Applicants believed that one of the main aims of selection should be to admit the best and most motivated students who best match the program and future profession (Figure 1). Applicants defined a fit not only as being equipped to become a successful student and future health professional, but also related this to the learning and working climate. Regarding a fit with the program, applicants believed that selection should contribute to reduced drop-outs and improved academic performance, but also to improved collaboration between students and a good atmosphere in the program.

When you do it right as a selection procedure is when you do well in terms of hard numbers, fewer drop-outs … higher student satisfaction, reduction of student stress that also helps tremendously, and when you take a broader view on diversity (I30).

With respect to selecting the best future health professionals, applicants also related this to both hard criteria (e.g., skills, efficiency), and softer criteria, such as improved job satisfaction.

That they just enjoy going to work more than just someone who already had no desire at all to take that degree and actually just barely made it. For example, someone who just does the work because they have to do it instead of really wanting to do it. And also, the more insightful doctors can have just a little bit more insight into the clinical picture of patients and possibly think of solutions in terms of medication or other things (I19).

#### Fulfilling societal needs

Applicants considered that selection should lead to cohorts of future health professionals who will contribute can fulfill societal needs, by improving quality of healthcare, increasing efficiency of healthcare and improving patient satisfaction. Examples of practical solutions included admitting students who have the ambition and skills to pursue a career in a specialty or geographic area with shortages, or students who can contribute to innovation in healthcare.

You can hear everywhere that there is an increasing shortage in certain areas of health care, there is also an immense improvement to be made in this regard … If you make a selection in Northern Netherlands, say Friesland, and there lives a whole group of students who very much want to become doctors and you use that in selection … then you can actually fill all those shortages (I11).

Applicants specifically mentioned that selection should generate a diverse student population. First, they believed diversity in personalities and qualities is important to meet the requirements for their future profession, as different qualities can be important depending on the specialty.

If my preferred selection would work, of course it’s not perfect, but the goal would be to get people with different skills and perspectives. This means diversity in healthcare, so people who are very good with patients in the social domain, but also many people who have a lot of knowledge and can apply it, diagnose and so on. And by working together and using these different perspectives, you can provide the best care for a patient (I13).‬‬‬

Second, diversity in background characteristics was believed to be necessary to generate a student population that is representative for the patient population they will serve. Applicants considered this important to meet societal needs, because, for instance, this would lead to more culturally sensitive care.

I think a whole range of people with different nationalities, diverse in gender … I do think that is important in the field. It is after all a reflection of the society. They are doctors, they have to deal with all layers of society. So, it is also very important, just as in politics, that it should be a reflection of society. I hope that that will be the result of my ideal selection procedure (I29).‬‬‬

### Contents of selection

#### Relevance

Applicants believed that it should be clear what attributes are necessary for the future profession, as well as for the curriculum, and align the selection procedure with these attributes. To effectuate this, selection committees should, according to applicants, define relevant attributes, and subsequently include instruments in their selection procedure that are relevant for the curriculum and the profession.

I don’t know what the curriculum looks like, but obviously the institute knows that, and I think they should select those who have attributes or have talent in the areas necessary for the study program (I27).

#### Comprehensiveness

Applicants also noted that selection committees are responsible for assessing a broad range of skills and attributes, thus that not only a subset of relevant skills is assessed, but all necessary attributes.

Yes, just assessing as many areas as possible … and if the selection committee would have all the time, then really interviewing people on different topics (I4).‬‬‬

When envisioning their ideal future, applicants also made comparisons with their current experience with selection. They addressed that current selection procedures are too narrow, as they mainly assess cognitive skills, and applicants thought that selection should focus more on the other necessary attributes.

Well actually I think the selection as it went down did fairly miss the mark. Everything I listed was in fact what they did not do there. It was a test of study material and a test of basic knowledge … Yes, they admit a selective group of students now, and by chance some of them may also be communicative and have other relevant skills, but they select only on intelligence now and I think that is a major flaw (I11).

#### Quality

Applicants were also concerned about the quality of the selection procedure. Applicants addressed the importance of valid and objective assessment, thus that instruments should assess what they intent to assess and there should be no room for confounding variables. Consequently, they believed that committees should be critical about which instruments are included.

The motivation letter at [university] was in the form of references from others, along with a letter in which the applicant reacts to the references. I think that is a good way to get a genuine impression of someone from their immediate surroundings. It includes both positive and less positive traits, which can provide insights into a person. I think if you write something yourself then you can often tailor it to what you think they want to hear. That is also my own experience ... However, others do not do that. They really write based on how they see you. (I26)‬‬‬

In addition to which instruments are included, applicants believed selection committees are responsible for other types of quality assurance to prevent bias, subjectivity, and social desirability, and to ensure consistency in administration.

We do have a problem with my ideal selection procedure, that is who is going to assess those students, of course. I do not think that it will be only one person, because one individual cannot interview at least 1200 students in such a short time. Then the problem will be that multiple people are assessing, and multiple people have different opinions, so there must be a protocol for these people to stick to (I8).

#### Distinctiveness

Applicants shared a clear desire to show who they are, what they can achieve, and, specifically, what differentiates them from others. According to applicants, distinction can best be based on non-cognitive skills, as those skills will make them ‘unique’. In addition, they believed distinction should be made on unlearnable attributes or talents that cannot be taught in the program. Although no consensus existed about what specifically entails unlearnable attributes, most applicants agreed that these mainly comprised non-cognitive skills.

I want to show that I am precise, critical, original, open minded. That I am motivated, obviously … Because I think that is what makes me unique. (I14)‬‬‬

Aside from assessing distinctive attributes, the instruments used to assess these attributes should also be able to differentiate. For example, they expressed a desire to raise the bar, as it will be very hard to differentiate on a selection instrument that is too easy.

Just a few questions that are really in-depth … That you know ahead of time that only 20% are going to answer this question correctly (I16).

#### Accommodation

Contrary to distinctiveness, applicants would also like the selection committee to accommodate to applicants’ developmental state. According to applicants, this would reduce the potential unequal impact of parental educational and occupational background on selection outcomes. Selection committees can achieve this by matching applicants’ current level and assessing minimal requirements, while providing room for them to further develop their skills and attributes over the course of the program. Additionally, they preferred a focus on current knowledge and skills, rather than on information about past achievement and behaviors, as they believed that their past did not define who they currently are.

Well of course there are certain skills that can be developed. I mean, you have to learn how to learn in a certain way. You learn that in secondary school as well, but that is also a different way of learning … You may have to learn the hard way sometimes and get a failing grade. Then you realize that you have to do things differently and I think you learn that very quickly during the training, especially if you are very motivated to study (I11).

Accommodation can be at odds with distinctiveness, at it is harder to distinguish between applicants when the bar is lower.

### Treatment of applicants

#### Reciprocity

According to applicants, selection should not only be an opportunity for the program to decide whether the applicant is suitable, but also the other way around. Therefore, they advocated for selection as a mutual process. They believed that during the selection procedure, applicants should get a realistic picture of the program. They would like to get to know the program’s contents, but also more procedural aspects such as study load.

I think it would be best if the program would also show a bit of what you are going to learn, what you are going to do. That they indirectly warn you that if this does not suit you, then maybe it is better not to enter the program (I22).

Another aspect that applicants highlighted is that selection should foster reflection upon their decision to apply. For instance, they shared a need for qualitative feedback on their selection outcomes to evaluate whether the program is suitable for them.

Giving feedback, because I think it is always good to improve yourself. So, suppose that pharmacy needs a different kind of student, then at least you know why you were not selected, and then you can take that into account the next time you apply (I1).

#### Well-being

According to applicants, selection committees are responsible for safeguarding their well-being. They mentioned that too much preparation can cause a lot of stress, because applicants need to combine multiple responsibilities, including graduating from secondary education.

Well, if you were to rely on a lottery system, there would be much less stress and nervousness. Currently, everyone is completely stressed out … You are constantly brooding in your head. I had that in the fifth year and part of the sixth year [pre-final and final year of pre-university education]. I was constantly thinking about it, this test, that school exam. It is a kind of chronic stress, because it is constant stress throughout the year (I29).

An additional threat to applicants’ well-being included competitiveness, which could in some cases result in applicants making major sacrifices, such as repeating a year to improve grades and taking a gap year to better prepare for the tests.

Applicants believed that their well-being can be improved through the treatment by the selection committee. They would like to receive a more personalized approach, in which they feel recognized as an individual.

I would definitely be very happy if it would be possible to also discuss a clinical case. Then, it is not just about looking at a piece of paper and saying, "Oh, this is just a name," but actually seeing the person. I think people would appreciate being invited for such an interview. It can be nerve-wracking for some, which is understandable. But I do believe that people would also feel relieved, knowing that they are not just a name or a student number. Yes, the selection process becomes much more personal (I23).

Other factors promoting well-being that were mentioned are positive communication and a welcoming environment.

It may be very personal … but just a piece of hope as it were. The letters that were sent to us tell us wat to do at what time, and that when you do not do it, you are not allowed to participate and you have actually already ruined it. That is the kind of vibe the letters we get have. I think this information should be provided, it should be clear what is expected, but what if they would also add the words “do your best” and emphasize that if you do not make it, it is not the end of the world. (I31).

At the same time, applicants acknowledged that their own preferred selection could result in reduced well-being, as many applicants preferred a combination of methods for comprehensiveness and found it important that applicants are prepared and committed.

#### Transparency

Applicants expressed a need for clear and transparent communication from the selection committee. They found it important that selection committees communicate (1) what applicants can expect during selection (e.g., what is assessed and when), and (2) what the program is looking for in students (e.g., what skills are important for a medical student, a realistic overview of the curriculum).

For pharmacy, I received some learning materials, and they did not ask anything about that at all in the test, maybe a few questions. I was also in the group chat of pharmacy and everybody was totally upset, in the Facebook group as well, so I thought oh I am not the only one. But it is weird: you are preparing for such a nerve-wrecking test and then you get such a weird test. They should just ask you about the study material (I1).

Additionally, applicants expressed a need to know what the program expects from applicants, for instance, which activities on a curriculum vitae (CV) will be valued over others. However, other applicants mentioned that such expectations should not be communicated towards applicants, as this may lead to applicants ‘gaming’ the system, creating a friction with quality.

I think it is good to have a selection procedure that is as vague as possible, so when you know as little as possible of what they are assessing. That is annoying for yourself, but I think in the end that it will prevent people from tweaking their CV or knowledge (I17).‬‬‬

#### Equality

Many applicants considered equal opportunities a core value in a fair selection procedure. They addressed that selection procedures should provide equal treatment to all applicants regardless of their educational or sociodemographic background.

Well first of all I think it is very important that everybody needs equal opportunities to begin with, in the sense that everyone should undergo the same selection procedure. Of course, voluntary work and extracurricular activities should be factored in. But right now, people with an 8 [on a scale from 1-10 with 10 as the highest] do not have to take the exam, they just get directly admitted (I31).‬‬‬

Moreover, according to applicants, selection committees should play an active role in reducing inequalities, not only by the design of the procedure (which instruments are included and how are they combined), but also with respect to access to information and resources that can help them perform on that procedure.

I think personality assessment is important, because if only tests are included in the second round – there are so many courses everywhere that really cost 150, 200 euros, and a lot of people have dads and moms who can pay that very nicely, but there are also plenty of people who cannot afford that or do not have the time for that (I23).

Applicants also recognized that a distinctive selection procedure which strives for excellence could be at odds with providing equal opportunities:

On one hand, I do think that it is unfair that if you decide later on what you want to study, you have less opportunity … But on the other hand, I think that many people do not know what they want to study and just send a motivation letter and CV, but just spent half an hour working on it. You do see the difference between those who really want it and take it seriously to work on a good CV and motivation letter, and I think that really identifies the most motivated students (I20).

#### Equity

Other applicants made a case for equity in selection: they believed that unequal treatment can be desirable to ensure equal outcomes. Applicants reckoned that many currently used instruments do not accurately represent applicants’ knowledge and skills, since performance is largely influenced by personal circumstances. Therefore, they preferred a more individual approach in which their performance is considered relative to their available time and resources.

I would take a look at everyone’s personal situation, I would not give that the most weight, certainly not, but I would combine that with grades and motivation … You can assess the personal circumstances by asking how much time they have available instead of only asking about how much time they spend on school … For example, if someone has 10 hours available and spends 10 hours studying, this may seem little compared to someone who has 40 hours and spends 30 hours studying (I19).

Another example of equitable treatment mentioned by applicants is that underrepresented groups should be admitted more often to enhance student diversity, and applicants believed that selection committees can take measurements to favor these groups of applicants over other groups.

### Translation from values into specific improvements

A complete overview of the specific improvements that applicants proposed regarding each value are collected in Table 2. For the value “finding a fit”, no specific improvements were mentioned, but instead applicants mentioned improvements related to relevance, comprehensiveness and reciprocity resulting in a better fit.

**Table 2:** Proposed improvements to selection procedures for each value

***Value***	***Proposed changes to the selection procedure***
Fulfilling societal needs	Include bonded medical places to address shortages in geographic areas or specialisms.
	Include lottery to improve student diversity.
Alignment	Assess attributes that are, according to applicants, relevant for becoming a successful student and future health professional: 1. Cognitive skills (e.g., intelligence, application of knowledge, logical reasoning, problem-solving skills, general knowledge, math skills, learning potential); 2. Personality characteristics (e.g., conscientiousness, empathy, adaptability, integrity, stress resistance); 3. Subject-specific knowledge and skills (prior knowledge, healthcare experience, clinical reasoning, research skills); 4. Motivation (e.g., intrinsic motivation, ambition, practical commitment, are applicants well-informed and well-prepared); 5. Social and communicative skills; 6. Planning and study skills
	Include instruments that assess attributes that are specifically relevant for the program and/or future profession. Examples of proposed instruments include: curriculum-sampling test, work sample (e.g., clinical case), simulation patient, curriculum vitae, group assignment, cognitive test, situational judgement test, interview, motivation letter, test of academic skills (e.g., test about scientific article).
	Ensure that instruments are of added value over the admission requirements and other instruments.
	Pay more attention to application of knowledge instead of reproduction in tests, as the former will be more important for the future profession.
	Involve current students in the development of the selection procedure, since they know what is important.
Comprehensiveness	Include a combination of instruments that assess different attributes.
	Specifically include additional instrument(s) that assess non-cognitive skills.
	Use a compensatory system to ensure that applicants with a combination of the necessary skills are selected.
	Ensure the weighting of cognitive and non-cognitive skills is balanced.
Quality	Include valid instruments that are thoroughly developed.
	Test the instruments prior to use to ensure that there are no mistakes in the instrument, and (in the case of a test) to ensure that applicants have enough time to finish.
	Take measures to prevent cheating and social desirability, especially with respect the assessment of motivation. Examples include: assess motivation during on-site selection days so applicants cannot get external help, include references in CV/motivation statements, and include video assessment of motivation.
	Take measures to prevent applicants from “gaming the system”. For example, assess extracurricular activities prior to study choice.
	Include multiple assessors in the selection committee that are trained and have multiple perspectives to reduce the risk of bias and subjectivity.
	Use standardized protocols for more subjective measures to prevent bias.
Distinctiveness	Include instruments on which applicants can really distinguish themselves. Examples include: personal file or portfolio, a pitch, a letter on which applicants describe their specific ambitions and goals.
	Pay attention to the format of instruments to ensure applicants have enough space to express themselves, such as a free format CV/portfolio, enough words in motivation letter.
	Include open-ended items in tests and registration forms.
	Raise the bar, for instance by including certain difficult test items that only the best few will successfully solve.
	Assess unlearnable attributes that cannot be taught during the program but are important for the future profession.
Entry-level	Ensure that the level of the assessment during selection matches applicants’ level, i.e., do not make assessment too difficult.
	Assess applicants based on their current state of attributes, and not on attributes of the past.
Reciprocity	Organize on-site selection days in order for applicants to get to know the campus and their peers.
	Include a curriculum-sampling test that adequately reflects the contents and study load of the curriculum.
	Provide preparatory and matching activities, including open days and walk-in days.
	Create a threshold for applicants to participate in the selection, for instance by requiring participation in a matching activity or a homework assignment.
	Provide qualitative feedback regarding the selection outcomes.
Well-being	Make sure that the amount of preparation time and materials do not intervene with applicants’ other responsibilities and well-being.
	Schedule the selection days outside of the exam weeks.
	Make sure the selection days are not too long, but also provide enough time for applicants to finish each test.
	Seek ways to be in personal contact with applicants, for instance by organizing on-site selection days and via personalized and positive communication.
Transparency	Communicate timely what is assessed, especially if applicants already need to put effort before applying (e.g., grades or CV).
	Provide explanation and/or opportunities to ask questions about the contents of the selection.
	Make sure that the contents of a test match the preparatory materials and information.
	Use a singular platform for communicating with applicants.
Equality	Make all applicants undergo the same selection procedure (thus no different tracks based on pre-university grades or prior education).
	Use a compensatory system so negative effects of certain instruments can be countered by other instruments.
	Include instruments that counter inequality, such as unweighted lottery.
	Make sure all applicants have access to information about the selection and to preparatory activities.
	Take measures in assessment to prevent unequal opportunities to perform. Examples include: assess motivation during on-site selection days so applicants cannot get external help, assess all kinds of work experience in a CV to counter unequal access to experience in the medical field, provide the preparatory materials for curriculum-sampling tests shortly before the exam to counter unequal preparation time.
Equity	Use contextualized admission to assess attributes in relation to the circumstances in which they were achieved.
	Include instruments that favor underrepresented groups.
	Include lottery with extra tickets for underrepresented groups.

## Discussion

The present study sought to gain a deeper understanding of applicant perceptions regarding values that should underlie selection into undergraduate HPE, and how these values can be used to make specific improvements to selection procedures. The findings of our study suggest that applicants believe that next to identifying the students who will be the best match with the curriculum and future profession, selection should also take the societal responsibility to generate a diverse student population that will contribute to improved quality of healthcare. Furthermore, applicants reported that the contents of the selection should be aligned with what is relevant for the curriculum and future profession, and a broad range of relevant factors should be taken into account, with valid instruments and a skilled committee. While applicants mostly expressed a desire to show what distinguishes them from others, they also believed that selection should focus on minimal requirements to provide room for growth. With respect to treatment of applicants, applicants valued a selection procedure that is a mutual process in which applicants are stimulated to reflect on their fit with the program. Additionally, applicants reported that selection committees are responsible for safeguarding applicant well-being, and transparent communication. Furthermore, applicants disagreed on whether selection committees should treat applicants equally or equitably. Finally, applicants provided specific suggestions for improvements to selection procedures. Although the findings are not always surprising, this study is, to our knowledge, the first to deconstruct applicant perceptions of the selection procedure in general rather than focusing on specific methods, by identifying applicants’ personal values with respect to selection.

**Fig. 2:**
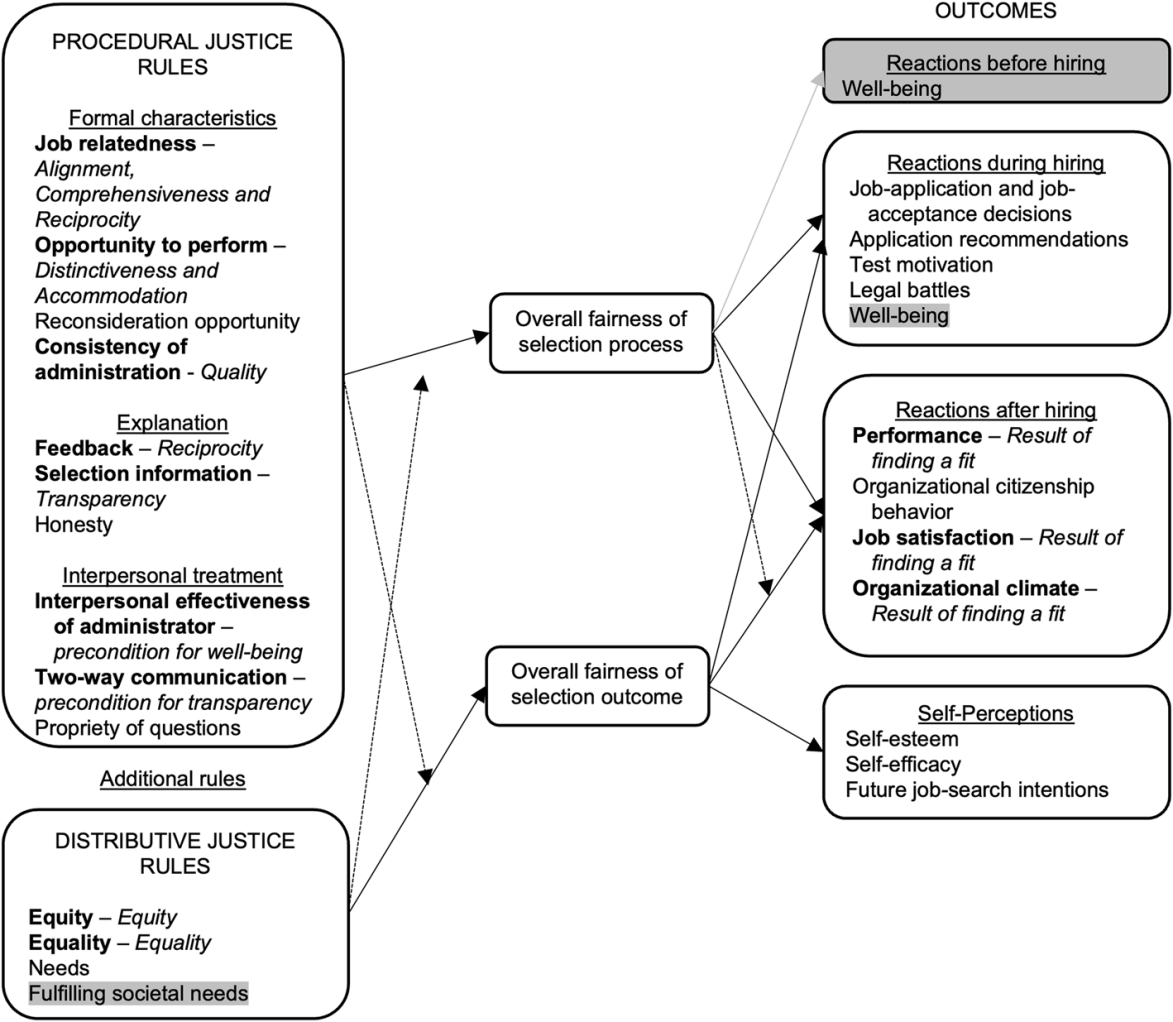
Comparison between Organizational Justice Theory and values underlying selection from the applicant perspective. *Legend.* The present study provides support and additions to the original conceptional model of organizational justice by Gilliland31. This figure shows the original model. Bold text indicates concepts of the original model that resonates with the concepts of the present study in Italic. Highlighted text indicates suggested additions to organizational justice theory

A theory that seems to resonate with the findings of the present study is organizational justice theory [25]. Organizational justice theory, commonly applied to describe applicant perceptions of selection, distinguishes between distributive justice, procedural justice, and outcomes of selection [25]. Distributive justice relates to the perceived fairness of the distribution of the outcomes of the selection procedures, while procedural justice describes the perceived fairness of the procedure used to generate this outcome. Finally, outcomes explain the attitudes and behaviors of applicants that are thought to be the result of perceptions regarding selection. Figure 2 depicts for which concepts of organizational justice theory we found support, as well as suggested additions to the theory, which will be discussed in the next sections.

Our study firstly adds to the literature by revealing applicants’ values with respect to distributive justice, an underreported justice dimension. Three values emerged regarding distributive justice, namely equity, equality and fulfilling societal needs. Although the former two are already described in organizational justice theory [27], our results suggest that the broader societal responsibility of selecting a (diverse) student population to fulfill societal needs can also be related to distributive justice. This goes beyond the concepts of equity and equality, as it relates to the distribution of outcomes based on what society needs rather than needs of individual applicants, and can be achieved through different measures, such as offering bonded medical places to combat shortages (as proposed by applicants and previous research [28]), emphasizing what the program values, and assessing necessary attributes [29]. Currently, efforts to equalize access to HPE are often described from a meritocratic stance with institutions providing opportunities to applicants (i.e., a deficit approach), while institutions generally do not acknowledge that increasing student diversity through selection can improve the quality of healthcare and education [30]. Indeed, selection procedures are often designed from the viewpoint of the institution, with its focus on improving academic performance and reducing drop-outs, while little attention is paid to the broader societal assignment of HPE [29, 31]. As applicants do acknowledge the societal responsibility of selection and the benefits of a diverse student population, it may be relevant to treat the societal impact of the outcomes of selection as a separate distributive justice component, at least within the field of HPE.

With respect to procedural justice, the present study uncovers which components are specifically relevant to applicants in undergraduate HPE. Procedural justice is usually divided into three components: formal characteristics of procedures, explanation of the procedures, and interpersonal treatment. Previous studies already reported on applicant perceptions of formal characteristics, that relate to the contents of selection, and results of the present study confirm these findings. For instance, the results of the present study suggest that applicants feel a desire to express and differentiate themselves (distinctiveness), which is in accordance with previous research [15, 32], and organizational justice theory [27]. Further research together with a diverse group of applicants is needed to identify appropriate methods that respond to this desire, as common methods such as written and verbal statements have their restrictions and may favor upper socio-economic groups with extensive networks to help them. Within HPE, research has paid much less attention to the other two procedural justice components [15], while it could be argued that the practical relevance of these components is even more evident as these aspects of selection are easier to adjust than the contents [33]. Our results suggest that applicants mainly consider the procedural justice dimension of explanation as important, as both the values of transparency and reciprocity can be related to this dimension [27]. With respect to transparency, research has shown that better explanation of selection procedures can improve perceptions of overall fairness [33, 34]. Nevertheless, although the importance of a transparent selection procedure has been acknowledged [35, 36], little is known about how transparent selection procedures in undergraduate HPE actually are to applicants. One study evaluating a selection procedure in life sciences found that even though applicants rated the selection they had undergone as moderately transparent, a large discrepancy existed between what applicants perceived to be important and the actual importance of selection criteria [37]. The value of reciprocity has elements in two procedural justice dimensions: according to applicants, selection should foster reflection on study choice by including methods that provide a realistic picture of the curriculum (i.e., formal characteristics) and providing feedback (i.e., explanation). Reciprocity has received some attention in research, for instance by the introduction of curriculum-sampling tests [38], and results of the present study reveal why applicants perceive this selection method favorably [15]. Likewise, situational judgements tests seem appropriate to provide information about the study program [39].

Finally, our study adds to the outcomes part of organizational justice theory. According to the theory, perceptions of selection can affect applicants’ outcomes during hiring, such as test-taking motivation and acceptance decisions. A previous study already found indications of an additional potentially relevant outcome, namely applicant well-being [15]. The results of the present study confirm this, as applicants again brought up feelings of stress and pressure as a negative outcome of selection. However, the present findings further suggest that applicant well-being should not only be considered as an outcome during hiring, but could even be impacted before hiring, as applicants noted that preparation for selection commences way before their application. As selection procedures – which can be considered assessment policy – are commonly designed from the perspective of the institution, little attention has been paid to the effects of their decisions on applicant well-being. For other assessment policies, some research has been conducted with this regard, and the results indicate that assessment policies can affect student well-being [40–42].

A strength of the present study is that we included applicants from a wide range of programs and from diverse backgrounds, reducing the risk of blind spots in our findings. However, a limitation related to the sampling is that participation in this study was based on a voluntary call-up, which may have resulted in a sample of relatively unsatisfied or opinionated applicants. A second limitation related to the sampling is the use of names to assume gender identity and ethnic background. Nevertheless, a wide range of perspectives and demographic backgrounds was represented in the results. Another limitation includes that applicants often struggled with expressing their values and would therefore often build upon specific examples or experiences rather than describing their values at an abstract level, which is probably related to the fact that the sample primarily included school leavers. The multiple perspectives and (professional) backgrounds in the research team assisted in handling ambiguous answers of applicants. A final limitation is that the findings are limited to the Dutch educational system. In selection procedures for jobs it has been suggested that applicants’ perceptions may depend both on personal attributes and on personal values tied to (national) cultures [43] and hence we invite researchers to assess if similar results can be found in other educational contexts.

The present study uncovered needs of applicants to selection in undergraduate HPE that remained unnoticed in prior research. A first step for future research could be to validate our selection values framework (Figure 1) and suggested additions to organizational justice theory (Figure 2) amongst applicants in different contexts. In addition, the present study could be replicated with other important stakeholder groups, including eligible high school students, applicants who dropped out of selection, selection committees and patients. This would provide insights into which values are widespread and which values are specific to certain stakeholder groups, and may also reveal potential blind spots of policy makers. Furthermore, more research is needed to find out how to reconcile high predictive validity with applicant perceptions. Previous studies already concluded that applicant perceptions can be at odds with other indicators of validity [11, 15], but the present study also revealed that applicants can recognize this themselves. Likewise, it would be interesting to further explore how to manage the frictions still found within applicant perceptions. For example, a Q-sort methodology could provide insights in the relative importance of conflicting values. Finally, as our results suggest that applicants desire a comprehensive selection procedure that predicts performance in the future profession, future research could pay more attention to translating the characteristics of a successful health professional (beyond existing competency frameworks )into competencies that can be measured at the start of the study program [44].

The present study provides numerous practical implications. Firstly, investigating what applicants find important in selection has provided concrete suggestions for improvement of selection procedures, in line with the underlying values of applicants. Notably, several aspects of selection desired by the applicants will be aspired to by most selection committees throughout the world. Unfortunately, these are not always reflected yet in the actual procedures. Moreover, compromises are often necessary. This study helps to understand where the preferences of candidates lie when compromises are required. Strikingly, the results of the present study suggest that previously reported contradictory applicant perceptions of selection methods [15] may be the result of conflicting values. This challenges selection committees to make a consideration of what they find important values, and thus create a clear vision on selection. Subsequently, committees should substantiate and communicate this vision towards applicants, and explain how applicants’ values are taken into account in this. Additionally, knowing what applicants value can provide insights in what programs should better explain to applicants in case their needs cannot be taken into account. Finally, our study indicates that it can be valuable to actively engage (prospective) students in the design of the selection procedure, as they are able to point out things that might be overlooked by selection committees.

## Conclusion

In conclusion, applicants believe that, in addition to being driven by institutional gains, selection in undergraduate HPE should generate a diverse student population to serve societal needs. Moreover, selection committees should equally pay attention to the contents or formal characteristics of selection, and to the treatment of applicants, specifically through providing better communication, fostering reflection upon study choice and taking well-being into account.

### Supplementary Information

Supplementary Material 1.

Supplementary Material 2.

## Data Availability

The data generated during the current study are not publicly available due to the sensitivity of the data, but are available from the corresponding author on reasonable request.

## Abbreviations

HPE: Health professions education
CV: Curriculum vitae

## References

[1] Kelly ME, Patterson F, O’Flynn S, Mulligan J, Murphy AW. A systematic review of stakeholder views of selection methods for medical schools admission. BMC Med Educ. 2018;18(1):1–26. 10.1186/S12909-018-1235-X. 29907112 10.1186/S12909-018-1235-X PMC6002997

[2] Stegers-Jager KM. Lessons learned from 15 years of non-grades-based selection for medical school. Med Educ. 2018;52(1):86–95. 10.1111/medu.13462. 28984374 10.1111/medu.13462 PMC5765503

[3] Patterson F, Knight A, Dowell J, Nicholson S, Cousans F, Cleland J. How effective are selection methods in medical education? A systematic review. Med Educ. 2016;50(1):36–60. 10.1111/medu.12817. 26695465 10.1111/medu.12817

[4] Feather NT. Values, valences, and choice: the influence of values on the perceived attractiveness and choice of alternatives. J Pers Soc Psychol. 1995;68(6):1135–51. 10.1037/0022-3514.68.6.1135. 10.1037/0022-3514.68.6.1135

[5] Schwartz SH. Values: cultural and individual. In: Fundamental Questions in Cross-Cultural Psychology. Cambridge University Press; 2012:463-493. 10.1017/cbo9780511974090.019

[6] Wouters A, Croiset G, Isik U, Kusurkar RA. Motivation of Dutch high school students from various backgrounds for applying to study medicine: A qualitative study. BMJ Open. 2017;7(5):e014779. 10.1136/bmjopen-2016-014779. 10.1136/bmjopen-2016-014779 PMC5623448 28576893

[7] Wouters A, Croiset G, Schripsema NR, et al. Students’ approaches to medical school choice: relationship with students’ characteristics and motivation. Int J Med Educ. 2017;8:217. 10.5116/IJME.5921.5090. 28624778 10.5116/IJME.5921.5090 PMC5511747

[8] Chan D, Schmitt N. Video-based versus paper-and-pencil method of assessment in situational judgment tests: Subgroup differences in test performance and face validity perceptions. J Appl Psychol. 1997;82(1):143–59. 10.1037/0021-9010.82.1.143. 9119795 10.1037/0021-9010.82.1.143

[9] Truxillo DM, Steiner DD, Gilliland SW. The importance of organizational justice in personnel selection: defining when selection fairness really matters. Int J Sel Assess. 2004;12(1–2):39–53. 10.1111/J.0965-075X.2004.00262.X. 10.1111/J.0965-075X.2004.00262.X

[10] Thorsteinson TJ, Ryan AM. The effect of selection ratio on perceptions of the fairness of a selection test battery. Int J Sel Assess. 1997;5(3):159–68. 10.1111/1468-2389.00056. 10.1111/1468-2389.00056

[11] Niessen ASM, Meijer RR, Tendeiro JN. Applying organizational justice theory to admission into higher education: Admission from a student perspective. Int J Sel Assess. 2017;25(1):72–84. 10.1111/ijsa.12161. 10.1111/ijsa.12161

[12] Greenhalgh T, Seyan K, Boynton P. “Not a university type”: focus group study of social class, ethnic, and sex differences in school pupils’ perceptions about medical school. BMJ. 2004;328(7455):1541. 10.1136/BMJ.328.7455.1541. 15217871 10.1136/BMJ.328.7455.1541 PMC437148

[13] Wouters A. Effects of medical school selection on student motivation: A PhD thesis report. Perspect Med Educ. 2018;7(1):54–7. 10.1007/s40037-017-0398-1. 29256053 10.1007/s40037-017-0398-1 PMC5807264

[14] Sheehan A, Thomson R, Arundell F, Pierce H. A mixed methods evaluation of multiple mini interviews for entry into the bachelor of midwifery. Women Birth. 2023;36(2):193–204. 10.1016/j.wombi.2022.08.005. 36050269 10.1016/j.wombi.2022.08.005

[15] Fikrat-Wevers S, Stegers-Jager K, Groenier M, et al. Applicant perceptions of selection methods for health professions education: rationales and subgroup differences. Med Educ. 2023;57(2):170–85. 10.1111/medu.14949. 36215062 10.1111/medu.14949 PMC10092456

[16] De Leng WE, Stegers-Jager KM, Born MP, Themmen APN. Influence of response instructions and response format on applicant perceptions of a situational judgement test for medical school selection. BMC Med Educ. 2018;18(1):1–10. 10.1186/s12909-018-1390-0. 30477494 10.1186/s12909-018-1390-0 PMC6258459

[17] Stegers-Jager KM, Themmen APN, Cohen-Schotanus J, Steyerberg EW. Predicting performance: relative importance of students’ background and past performance. Med Educ. 2015;49(9):933–45. 10.1111/MEDU.12779. 26296410 10.1111/MEDU.12779

[18] Martin AJ, Beska BJ, Wood G, et al. Widening interest, widening participation: factors influencing school students’ aspirations to study medicine. BMC Med Educ. 2018;18(1):1–13. 10.1186/S12909-018-1221-3. 29843689 10.1186/S12909-018-1221-3 PMC5975409

[19] Bunniss S, Kelly DR. Research paradigms in medical education research. Med Educ. 2010;44(4):358–66. 10.1111/J.1365-2923.2009.03611.X. 20444071 10.1111/J.1365-2923.2009.03611.X

[20] Niessen AS, Meijer RR. Selection of medical students on the basis of non-academic skills: is it worth the trouble? Clin Med. 2016;16(4):339–42. 10.7861/clinmedicine.16-4-339. 10.7861/clinmedicine.16-4-339 PMC6280215 27481377

[21] Fikrat-Wevers S, Stegers-Jager KM, Afonso PM, et al. Selection tools and student diversity in health professions education: a multi-site study. Adv Heal Sci Educ. 2023:1-26. 10.1007/S10459-022-10204-9/TABLES/9 10.1007/s10459-022-10204-9 PMC9848043 36653557

[22] Mulder L, Wouters A, Fikrat-Wevers S, et al. Influence of social networks in healthcare on preparation for selection procedures of health professions education: a Dutch interview study. BMJ Open. 2022;12(10):e062474. 10.1136/BMJOPEN-2022-062474. 10.1136/BMJOPEN-2022-062474 PMC9628659 36316069

[23] Varpio L, Ajjawi R, Monrouxe LV, O’Brien BC, Rees CE. Shedding the cobra effect: problematising thematic emergence, triangulation, saturation and member checking. Med Educ. 2017;51(1):40–50. 10.1111/MEDU.13124. 27981658 10.1111/MEDU.13124

[24] Sandars J, Murdoch-Eaton D. Appreciative inquiry in medical education. Med Teach. 2017;39(2):123–7. 10.1080/0142159X.2017.1245852. 27852144 10.1080/0142159X.2017.1245852

[25] Hsieh HF, Shannon SE. Three approaches to qualitative content analysis. Qual Health Res. 2005;15(9):1277–88. 10.1177/1049732305276687. 16204405 10.1177/1049732305276687

[26] Kiger ME, Varpio L. Thematic analysis of qualitative data: AMEE Guide No. 131. Med Teach. 2020;42(8):846–54. 10.1080/0142159X.2020.1755030. 32356468 10.1080/0142159X.2020.1755030

[27] Gilliland SW. The perceived fairness of selection systems: an organizational justice perspective. Acad Manag Rev. 1993;18(4):694–734. 10.5465/amr.1993.9402210155. 10.5465/amr.1993.9402210155

[28] Puddey IB, Mercer A, Carr SE, Louden W. Potential influence of selection criteria on the demographic composition of students in an Australian medical school. BMC Med Educ. 2011;11(1):1–12. 10.1186/1472-6920-11-97. 22111521 10.1186/1472-6920-11-97 PMC3233506

[29] Wouters A, Croiset G, Kusurkar RA. Selection and lottery in medical school admissions: who gains and who loses? MedEdPublish. 2018;7(4):271. 10.15694/mep.2018.0000271.1. 38089206 10.15694/mep.2018.0000271.1 PMC10711990

[30] Alexander K, Fahey Palma T, Nicholson S, Cleland J. “Why not you?” Discourses of widening access on UK medical school websites. Med Educ. 2017;51(6):598–611. 10.1111/MEDU.13264. 28229477 10.1111/MEDU.13264

[31] Inspectie van het Onderwijs. Selectie in Het Hoger Onderwijs: Criteria, Instrumenten En de Borging van Kansengelijkheid; 2023.

[32] White JS, Lemay JF, Brownell K, Lockyer J. “A chance to show yourself” - How do applicants approach medical school admission essays? Med Teach. 2011;33(10):e541. 10.3109/0142159X.2011.599890. 21942490 10.3109/0142159X.2011.599890

[33] Truxillo DM, Bodner TE, Bertolino M, Bauer TN, Yonce CA. Effects of explanations on applicant reactions: a meta-analytic review. Int J Sel Assess. 2009;17(4):346–61. 10.1111/j.1468-2389.2009.00478.x. 10.1111/j.1468-2389.2009.00478.x

[34] Patterson F, Zibarras L, Carr V, Irish B, Gregory S. Evaluating candidate reactions to selection practices using organisational justice theory. Med Educ. 2011;45(3):289–97. 10.1111/J.1365-2923.2010.03808.X. 21299603 10.1111/J.1365-2923.2010.03808.X

[35] Patterson F, Roberts C, Hanson MD, et al. 2018 Ottawa consensus statement: Selection and recruitment to the healthcare professions. Med Teach. 2018;40(11):1091–101. 10.1080/0142159X.2018.1498589. 30251906 10.1080/0142159X.2018.1498589

[36] Patterson F, Lievens F, Kerrin M, Zibarras L, Carette B. Designing selection systems for medicine: the importance of balancing predictive and political validity in high-stakes selection contexts. Int J Sel Assess. 2012;20(4):486–96. 10.1111/IJSA.12011. 10.1111/IJSA.12011

[37] Kurysheva A, van Rijen HVM, Dilaver G. How do admission committees select? Do applicants know how they select? Selection criteria and transparency at a Dutch University. Tert Educ Manag. 2019;25(4):367–88. 10.1007/S11233-019-09050-Z/FIGURES/3. 10.1007/S11233-019-09050-Z/FIGURES/3

[38] Niessen ASM, Meijer RR, Tendeiro JN. Admission testing for higher education: A multi-cohort study on the validity of high-fidelity curriculum-sampling tests. Plos One. 2018;13(6):e0198746. 10.1371/journal.pone.0198746. 29889898 10.1371/journal.pone.0198746 PMC5995396

[39] Fröhlich M, Kahmann J, Kadmon M. Development and psychometric examination of a German video-based situational judgment test for social competencies in medical school applicants. Int J Sel Assess. 2017;25(1):94–110. 10.1111/IJSA.12163. 10.1111/IJSA.12163

[40] Lyndon MP, Strom JM, Alyami HM, et al. The relationship between academic assessment and psychological distress among medical students: a systematic review. Perspect Med Educ. 2014;3(6):405–18. 10.1007/S40037-014-0148-6/FIGURES/2. 25428333 10.1007/S40037-014-0148-6/FIGURES/2 PMC4263790

[41] Stegers-Jager KM, Savas M, van der Waal J, van Rossum EFC, Woltman AM. Gender-specific effects of raising Year-1 standards on medical students’ academic performance and stress levels. Med Educ. 2020;54(6):538–46. 10.1111/MEDU.14068. 31960979 10.1111/MEDU.14068 PMC7317944

[42] Wasson LT, Cusmano A, Meli L, et al. Association between learning environment interventions and medical student well-being: a systematic review. JAMA. 2016;316(21):2237–52. 10.1001/JAMA.2016.17573. 27923091 10.1001/JAMA.2016.17573 PMC5240821

[43] Wang Q, Hackett RD, Zhang Y, Cui X. Personal characteristics and applicants’ perceptions of procedural fairness in a selection context: The mediating role of procedural fairness expectations. Manage Decis. 2020;58(4):687–704. 10.1108/MD-01-2018-0088. 10.1108/MD-01-2018-0088

[44] Born MP, Stegers-Jager KM, Van Andel CEE, Mc E. Inferring signs from purposeful samples: The role of context in competency assessment. Med Educ. 2021;56:117. 10.1111/medu.14669. 34558107 10.1111/medu.14669 PMC9293475

