# Supplementary material for: Improving selection procedures in health professions education from the applicant perspective: an interview study
Source: BMC Med Educ. 2024 Aug 7;24:849. doi: 10.1186/s12909-024-05761-z (PMC11308236; doi:10.1186/s12909-024-05761-z)
Supplement: Supplementary file 1 — Supplementary Material 1. [file 12909_2024_5761_MOESM1_ESM.pdf]

Additional file 1. Selection procedures of the five programs included in the present study

Program*	Type	Selection procedure	Weighting
Amsterdam UMC, location AMC	Medicine	Procedure included only a testing day with the following tests:<br>• **Curriculum-sampling test.** Multiple-choice test based on online study materials (texts, video lecture, knowledge clips).<br>• **Biomedical knowledge test.** Multiple-choice test on the application of biomedical knowledge typically covered in pre-university education, and on news topics in the medical field.<br>• **Medical ethical essay test.** Writing task of three short essays to reflect on medical ethical dilemmas. This test only counted for 150 applicants with an identical sum score on the other two tests and scoring around the threshold for these tests.	 50%<br>50%<br><br>0%/decisive in the event of a tie
Erasmus MC	Medicine	Procedure included an entry form including the following:<br>• **Pre-GPA.** Based on the grades for Dutch and English language, mathematics, physics, science, and biology. For applicants not directly applying from pre-university education (i.e., who graduated in a previous year or applied from an alternative type of education), pu-GPA wasn't included and the other two criteria both weighted 50%.<br>• **CV.** Included work experience, board position, or exceptional performance in the areas of science, arts, literature or sports. Applicants were assessed based on their argumentation and not the activity itself.<br>An on-site testing day which included the following tests:<br>• **Curriculum-sampling test.** Test including both multiple choice and open items, based on a lecture<br>• **Math test:** mathematical items with relevant for medicine<br>• Academic reading test: MC test with one open question on getting the essence of several scientific articles: .	 33%<br><br><br>33%<br><br><br><br>33%
Amsterdam UMC, location VUmc	Medicine	**Round 1** included an entry form, on which a preselection was made. A composite score was calculated on:<br>• **Prior education.** Scores based on total grades list and potential additional extracurricular education.<br>• **CV.** Included work experience, board position, experience in technical science or research, or exceptional performance in the areas of arts, literature or sports.<br>**Round 2** included a testing day, including the following tests:<br>• **Curriculum-sampling test.** Test based on a lecture and online study materials.<br>• **Cognitive capacity test.** Assessed skills included verbal reasoning skills, numerical reasoning ability, spatial awareness and Dutch language proficiency.<br>This program distinguished between applicants based on educational background. Applicants who weren't directly applying pre-university education (i.e., who graduated in a previous year or applied from an alternative type of education), underwent the same procedure as those who were, but received different scoring as a quotum was set in place (i.e., a maximum of 30% of the selected students could	 50%<br><br><br><br>50%
University of Twente	Technical Medicine	Procedure included an entry form including the following:<br>• **Pre-GPA.** Based on the grades for mathematics, physics, biology, and overall pu-GPA.<br>The BMAT was administered during an on-site testing day:<br>• **Aptitude and skills test (BMAT 1).** Multiple-choice test on general skills in problem solving, inferences, and data analysis.<br>• **Biomedical knowledge test (BMAT 2).** Multiple-choice test on applying scientific knowledge from biomedical subjects covered in pre-university education.<br>• **Writing task (BMAT 3).** Task on the ability to develop and organize ideas, and to communicate them in writing.	 60%<br><br>10%<br>20%<br><br>10%
Utrecht University	Pharmacy	Procedure included an entry form including the following:<br>• **Pe-GPA.** Based on the grades for mathematics, physics, biology, and overall pu-GPA.<br>The following tests were administered during an a testing day:<br>• **Curriculum-sampling test.** Open questions test based on a bundle of articles, including mathematical items.<br>• **Curriculum-sampling test (short-term).** Open questions test based on a lecture and problem-based learning tutorial that took place during the testing day.	 40%<br><br>30%<br>30%

Note. Pre-GPA = prior education grade point average; CV = curriculum vitae; BMAT = BioMedical Admissions Test.
