# Supplementary material for: Improving selection procedures in health professions education from the applicant perspective: an interview study
Source: BMC Med Educ. 2024 Aug 7;24:849. doi: 10.1186/s12909-024-05761-z (PMC11308236; doi:10.1186/s12909-024-05761-z)
Supplement: Supplementary file 2 — Supplementary Material 2. [file 12909_2024_5761_MOESM2_ESM.pdf]

Additional file 2: topic list

1. **Tell me something about an experience in which you were selected or chosen to perform something you are good at.**
    - Examples: selected for a job or internship position, selected for a certain committee or representative function, a sports team.
    - In which context did this situation take place?
    - What exactly are you good at? What do you have to offer that makes you suitable to perform the activity for which you were selected?
    - What did you exactly display in this situation? What knowledge, skills, experience or personality characteristics were you able to show?
    - To whom did you demonstrate these things? What did they do that enabled you to demonstrate these things?
    - How did you feel at that moment? And what feeling stands out when you remember the experience?
    - What is the main point of your story?

2. **What values underly a proper selection procedure, in your opinion?**
    - From what conviction should the study program design its selection procedure, and why?
    - What do you already need to be able to perform to be admitted to the study program? What skills or knowledge should you be allowed to develop during the study program?
    - When is the selection procedure successful?
        i. What are you able to show?
        ii. Who are selected, and why? Why is this beneficial for future health care?
    - When is the selection procedure unsuccessful?
        i. What can you not show that is important to show?
        ii. What do you have to show that is not important at all?
        iii. Who will be missed that may be potential successful students?
        iv. What goes wrong during education and after graduating?
    - What is the main point of your story?

3. **Imagine it is 2022. The first students are selected in a new way. Imagine that they are selected according to your ideal selection procedure.**
    - What difference has been made?
    - Who are walking around the campus?
    - What knowledge, skills and personality aspects are assessed in this selection procedure?
    - Which (combinations of) selection instruments are used?
    - How is achieved that the “right” students are attracted to the program?
    - What will be the effect of your ideal selection procedure on future applicants who are preparing for the selection procedure?
    - What will be the effect of your ideal selection procedure on the experience of admitted students?
    - What will be the effect of your ideal selection procedure on the people who graduate the program? What will it contribute to society and health care?
    - Are there any other requirements for your ideal selection procedure?

4. **Imagine you are allowed to change three things about the selection procedure of the program to which you applied to. What would those three things be?**
